# Primary tooth pulpectomy overfilling by different placement techniques: A systematic review and meta-analysis

**DOI:** 10.34172/joddd.2020.043

**Published:** 2020-11-26

**Authors:** Naser Asl Aminabadi, Nahid Asl Aminabadi, Zahra Jamali, Sajjad Shirazi

**Affiliations:** ^1^Department of Pediatric Dentistry, Faculty of Dentistry, Tabriz University of Medical Science, Tabriz, Iran; ^2^Department of Oral and Maxillofacial Radiology, Faculty of Dentistry, Tabriz University of Medical Sciences, Tabriz, Iran; ^3^Department of Oral Medicine, Faculty of Dentistry, Tabriz University of Medical Sciences, Tabriz, Iran; ^4^Department of Oral Biology, College of Dentistry, University of Illinois at Chicago, Chicago, IL, USA

**Keywords:** Child, Deciduous, Preschool, Pulpectomy, Root canal, Root canal therapy, Tooth

## Abstract

**Background.** This study was conducted to investigate root canal overfilling with different material placement techniques in primary teeth.

**Methods.** A systematic search was undertaken by searching PubMed/MEDLINE and Scopus for English language peer-reviewed articles published until February 2018 that reported primary tooth pulpectomy overfilling. Two reviewers independently screened and identified studies in terms of the selection criteria and independently collected the data using a specially designed data extraction form. The overfilling rate was the primary summary measure. The weighted pooled overfilling rates were estimated by random-effects meta-analysis.

**Results.** Twenty clinical and four in vitro studies met the eligibility criteria. In the clinical studies, the pooled overfilling rate for zinc oxide-eugenol (ZOE) was 23.3% with a lentulo spiral mounted on a handpiece, 22.7% with a hand-held lentulo spiral, and 17% with a plugger. The pooled overfilling rate for calcium hydroxide-based materials was 16.7% with a lentulo spiral mounted on a handpiece, 14.7% with a hand-held lentulo spiral, 19.6% with a syringe, and 25.7% with a plugger. In the in vitro studies, neither individual overfilling rates nor two-by-two comparisons were subjected to meta-analysis because of an inadequate number of studies.

**Conclusion.** The lowest overfilling rate in the clinical studies was related to plugger and handheld lentulo spiral techniques for ZOE and calcium hydroxide-based materials, respectively

## Introduction

Pulpectomy of primary teeth is indicated when the pulp tissue is irreversibly infected or necrotic due to caries or trauma. The treatment consists of extirpation of the pulp tissue, removal of organic debris with filing, and obturation of the canals with a suitable material.^1^ Obturation with an optimum length, minimum voids, and a hermetic seal are necessary for successful endodontic treatment in primary teeth. However, the complexity of the root canal system and its resorption pattern in primary teeth might interfere with the ideal filling of the canal.^2-4^

It has been noted that the success of pulpectomies with adequate or short fills is significantly higher than those with overfilling.^5,6^ Potential drawbacks of overfilling are foreign body reaction,^6^ arrested formation,^7^ and deflection of the eruption path of the succedaneous tooth.^8,9^ Enamel defects in succedaneous teeth might be observed when there are extensive preoperative root resorption and a long fill approximating the developing tooth’s crypt.^5^ Zinc oxide-eugenol (ZOE) is a moderately resorbable material, and unresorbed ZOE has been reported in pulpectomized primary teeth with overfilling in the long term^8,10-13^ and after exfoliation.^8,12-15^ On the other hand, complete resorption of extruded calcium hydroxide-based materials has been reported in almost 100% of overfilled cases.^11,16,17^

Overall, it seems that root canal overfilling is associated with greater risk than normal and underfilling. Although various techniques have been used for root canal filling in primary teeth, previous findings regarding the effectiveness of these techniques for adequate filling of root canals have yielded controversial results, with no consensus about one particular technique’s superiority.^3,18-25^ Therefore, this systematic review was conducted to explore the overfilling rate with different root canal filling techniques in primary teeth in the available clinical and in vitro studies.

## Methods

This study was conducted according to the Preferred Reporting Items for Systematic Review and Meta-Analysis (PRISMA).

### Search strategy and study identification

A systematic search was conducted by a professional librarian with skills in informatics by searching the electronic databases PubMed/MEDLINE and Scopus for English language peer-reviewed articles published until February 2018 using the following search strategy (“root canal filling” OR “root canal obturation” OR “root canal obturating” OR “root canal treatment” OR “root canal therapy” OR “obturation method” OR “obturation methods” OR “obturation technique” OR “obturation techniques” OR “obturation” OR “obturating” OR “pulp therapy” OR “pulpectomy”) AND (“child” OR “children” OR “deciduous” OR “primary teeth” OR “primary tooth” OR “primary molar”).

After searching the databases, some prestigious journals in this field, including the International Journal of Paediatric Dentistry, Pediatric Dentistry, The Journal of Clinical Pediatric Dentistry, European Archives of Paediatric Dentistry, Journal of Dentistry for Children, International Endodontic Journal and Journal of Endodontics, were also hand-searched. In addition, the reference lists of selected articles were manually searched to complete the search database. We also scanned the Cochrane database and reference lists from review articles identified in the searches for further studies and consulted reference lists from pediatric dentistry textbooks. A database was created for the found records, where duplicate entries were removed.

### Eligibility criteria

Criteria for considering studies for this review were as follows: Clinical study (RCT, cross-sectional, prospective, etc.) or in vitro study on primary tooth root canal treatment; abstract available in English; complete root or remaining root length of two-third or more; the frequency of root canal overfilling (determined immediately after each treatment through radiographs) in treatment groups that were given or could be calculated from the raw data; sample size given for each group within the study; the technique used for root canal filling mentioned; the working length from the apex and the size of the last file used for the root canal instrumentation specified.

Case reports, review articles, editorials, opinions, technique articles, surveys, guidelines, and commentary articles were excluded.

### Data collection

The initial selection was based on the titles and abstracts of the obtained studies. Two reviewers independently screened and identified studies in terms of the selection criteria. Whenever the fulfillment of these criteria was not clear from the abstract, the study’s full text was obtained for verification. Disagreements on study inclusion were resolved by discussion. All the papers that passed the abstract screening were retrieved in their complete forms, and data extraction was conducted. The reasons for study exclusion were recorded at this stage or subsequent stages.

The two reviewers independently collected data using a specially designed data extraction form, which was pilot-tested with 10 articles and modified as required before use. The data presented in graphs and figures were extracted whenever possible but included only if both reviewers independently had the same result or the study authors could provide clarification of data. Disagreements at any stage were resolved by discussion.

The following data were recorded for each study: year of publication and country of origin; study design; a detailed description of root canal instrumentation, including file size and type, working length from the apex, filling material, tooth type and operator; radiographic criteria of the extent of root canal filling, number of radiographic assessors and calculation of inter-examiner reliability; unit of outcome measure (tooth or canal), sample size and number of overfilled canals or teeth as determined immediately after each treatment through radiographs. The overfilling rate was the primary summary measure.

### Assessment of risk of bias for each included study

Two reviewers independently summarized the risk of bias for the outcome within each included study according to the domain-based evaluation described in the *Cochrane Handbook for Systematic Reviews of Interventions 5.1.0*.^26^ The following domains were assessed: generation of allocation sequence, allocation concealment, blinding of radiological outcome assessors, and missing data. Selective outcome reporting was not assessed because no study protocol or registration was accessible. Blinding of personnel (performance bias) was also not assessed, considering that different techniques were used. Attrition bias was not assessed since the assessment of overfilling in the included studies was undertaken only at baseline immediately after each treatment.

The overall risk of bias within each study was classified as “low” risk of bias (a plausible bias unlikely to seriously alter the results) if all the above criteria were met; “unclear” risk of bias (a plausible bias that raises some doubt about the results) if one or more criteria were assessed as unclear; or “high” risk of bias (plausible bias that seriously weakens confidence in the results) if one or more criteria were not met.

### Data synthesis

CMA version 2.2 statistical software was used to perform all statistical analyses. Only categories with three or more studies were included in the final meta-analysis. The unit of analysis was either tooth or canal. Forest plots, Cochran’s (Q) test, and I^2^ coefficient were used to investigate statistical heterogeneity. The I^2^ statistic was used with an approximate guide for interpretation as follows: 0‒40%, not important heterogeneity; 40‒60%, moderate heterogeneity, and 60‒90%, substantial heterogeneity.^26^ Unweighted overfilling rate of different techniques within each study was calculated by dividing the total number of outcome units (overfilled root or canal) by the total number of units (root or canal) within the respective technique category at 95% confidence intervals (CIs). In addition, the relative weights of overfilling rates within each technique category were calculated. The weighted pooled overfilling rate for each technique category was estimated by random-effects meta-analysis.

## Results

### Description of studies

**Figure 1:**
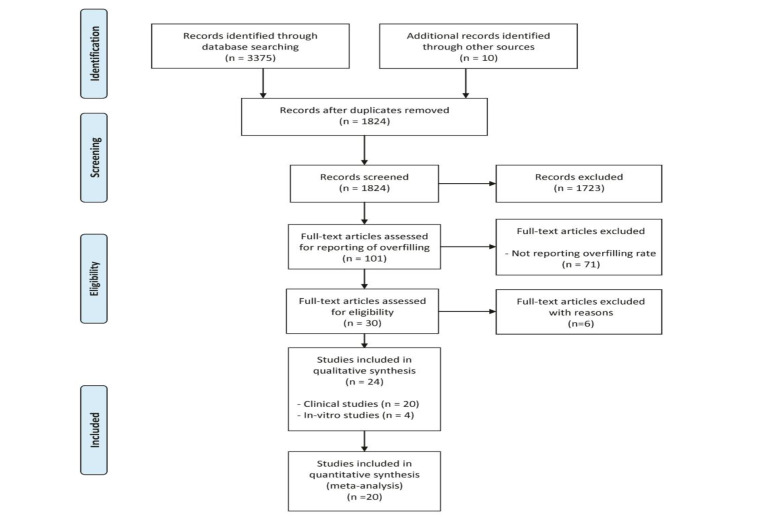
Literature review flow diagram.

Initial searches from all the sources identified 1824 unique references. After scanning the titles and abstracts, the full texts of 101 studies were obtained, and data extraction was performed. Seventy-one studies were excluded for not reporting overfilling. Six studies were excluded for not satisfying the review inclusion criteria. Twenty clinical and four in vitro studies satisfied the eligibility criteria for the review (Figure 1). Only six of the clinical studies explicitly aimed at comparing different obturation techniques.^18-20,27-29^ All the included in vitro studies were conducted to compare different obturation methods.^2,3,30,31^ Of the 20 included clinical studies, 16 were randomized clinical trials, and four were cross-sectional. The year of publication was from 1993 to 2017. The studies were conducted in India (n=13), Iran (n=2), United States (n=1), Brazil (n=2), Thailand (n=2), UAE (n=1), Turkey (n=2), and Saudi Arabia (n=1). The full description and characteristics of the included studies are presented in Table 1.

**Table 1:** Full description and characteristics of the included clinical studies for ZOE

**Study**	**Country**	**Material**	**Technique**	**Operator**	**Last file size**	**File type**	**N**	**Overfill**	**Unit of outcome**	**Tooth type**	**Filing distance from apex**	**Study** **Design**	**Radiographic assessors**	**Inter-examiner reliability**
Ann Mani, 2000^46^	India	ZOE	Lentulo	NI	35	H	60	15	Canal	Molar	1	Cross-sectional	NI	
Barcelos, 2011^32^	Brazil	ZOE	Handpiece lentulo	Postgraduate student	35	K	67	14	Tooth	Molar, incisor	1	RCT	double-blinded and calibrated	Yes
Bawazir, 2005^20^	Saudi Arabia	ZOE	Handpiece lentulo	NI	35, 50	H	25	4	Tooth	Molar	1	RCT	double-blinded	
			Lentulo				25	2						
Damle, 2005^47^	India	ZOE	Pressure syringe	NI	40	H	35	4	Tooth	Molar	1	RCT	NI	
Gupta, 2011^48^	India	ZOE	Plugger with cotton pellet	NI	30	H	21	2	Tooth	Molar	1-2	Cross-sectional	NI	
Khubchandani, 2017^29^	India	ZOE	Handpiece lentulo	NI	35	H	30	8	Canal	Molar	1	RCT	triple-blinded	No
			Navitip				30	15						
Mortazavi, 2004^11^	Iran	ZOE	Plugger with cotton pellet	NI	30	K	32	7	Tooth	Molar, incisor	1-2	RCT	NI	
Ozalp, 2005^17^	Turkey	ZOE	Lentulo	Pediatric Dentist	30-35	H	20	6	Tooth	Molar	1	RCT	NI	
Ramar, 2010^49^	India	ZOE	Handpiece lentulo	NI	35	H	68	12	Canal	Molar	1-2	RCT	NI	
Rewal, 2014^50^	India	ZOE	Handpiece lentulo	Pediatric Dentist	35	H	24	10	Tooth	Molar	1-2	RCT	double- calibrated	Yes
Subramaniam, 2011^40^	India	ZOE	Plugger with cotton pellet	NI	35	H	15	2	Tooth	Molar	1	Cross-sectional	NI	
Tannure, 2010^51^	Brazil	ZOE	Handpiece lentulo	Pediatric Dentist	35	K	36	9	Tooth	Incisor	1	RCT	One	
Vashista, 2015^19^	India	ZOE	Lentulo	NI	25-30	K	30	6	Tooth	Molar, incisor	1	Cross-sectional	Double-blinded	Yes
			Pressure syringe				30	11						

ZOE: Zinc-Oxide Eugenol; NI: not indicated; RCT: randomized clinical trial.

In the clinical studies, nine different techniques, including a hand-held lentulo spiral, plugger with a cotton pellet, bi-directional spiral, past inject, pressure syringe, Navitip, provided syringe, lentulo spiral mounted on a handpiece, and disposable syringe were used for the root canal obturation. Six different materials were used for root canal filling, categorized into ZOE and calcium hydroxide-based materials, including Vitapex, Sealapex, Metapex, Endoflas, and calcium hydroxide itself (Tables 1 and 2).

**Table 2:** Full description and characteristics of the included clinical studies for Calcium hydroxide-based materials

**Study**	**Country**	**Material**	**Technique**	**Operator**	**Last file size**	**File type**	**N**	**Overfill**	**Unit of outcome**	**Tooth type**	**Filing distance from apex (mm)**	**Study** **Design**	**Radiographic assessors**	**Inter-examiner reliability**
Ann Mani, 2000^46^	India	Calcium hydroxide	Lentulo	NI	30-35	H	60	11	Canal	Molar	1	cross-sectional	NI	
Chawala, 2008^10^	India	Calcium hydroxide	Lentulo	Postgraduate student	35	H	25	3	Tooth	Molar	1	cross-sectional	NI	
Damle, 2005^47^	India	Calcium hydroxide	Pressure syringe	NI	40	H	35	4	Tooth	Molar	1	RCT	NI	
Grover, 2013^18^	India	Endoflas	Handpiece lentulo	NI	30-35	H	28	3	Canal	Molar, Incisor	1	RCT	Double-blinded	
			Pastinject				28	4						
Pressure syringe	28	22
Bi-directional spiral	28	0
Gandhi, 2017^28^	India	Endoflas	Disposable syringe	NI	30-35	H	20	6	Tooth	Molar	1	RCT	Double-blinded	
			Lentulo				20	2						
Pastinject	20	2
Gupta, 2011^48^	India	Metapex	Provided syringe	NI	30	H	21	3	Tooth	Molar	2-1	Cross-sectional	NI	
Louwakul, 2012^52^	Thailand	Vitapex	Provided syringe	Pediatric Dentist	35-40	K	60	15	Tooth	Molar	1	RCT	Double-blinded and calibrated	Yes
Mortazavi, 2004^11^	Iran	Vitapex	Plugger with cotton pellet	NI	30	K	26	10	Tooth	Molar, incisor	2-1	RCT	NI	
Nakornchai, 2010^53^	Thailand	Vitapex	Provided syringe	NI	25-30	K	25	19	Tooth	Molar	2-3	RCT	Double-calibrated	Yes
Ozalp, 2005^17^	Turkey	Sealapex	Lentulo	Pediatric Dentist	35	H	20	0	Tooth	Molar	1	RCT	NI	
		Vitapex	Provided syringe				40	7						
Pandranki, 2017^27^	India	Endoflas	Plugger with cotton pellet	NI	35	K	15	3	Tooth	Molar	1	RCT	Double-blinded and calibrated	Yes
			Handpiece lentulo				45	4	Canal					
			Navitip				45	10						
Ramar, 2010^49^	India	Endoflas	Handpiece lentulo	NI	35	H	64	11	Canal	Molar	1-2	RCT	NI	
		Metapex	Provided syringe				30	2	Tooth					
Rewal, 2014^50^	India	Endoflas	Handpiece lentulo	Pediatric Dentist	35	H	52	12	Canal	Molar	2-1	RCT	Double-calibrated	Yes
Sarý, 2008^54^	Turkey	Sealapex	Handpiece lentulo	NI	40	H	52	19	Tooth	Molar, incisor	1	Cross-sectional	One	
Subramaniam, 2011^40^	India	Endoflas	Plugger with cotton pellet	NI	35	H	15	2	Tooth	Molar	1-2	RCT	NI	
		Metapex	Provided syringe				15	3						

NI: not indicated; RCT: randomized clinical trial.

In the in vitro studies, the root canals were filled with ZOE or Vitapex using 10 different techniques, including a hand-held lentulo spiral, plugger with a cotton pellet, pressure syringe, lentulo spiral mounted on a handpiece, tuberculin syringe, insulin syringe, provided syringe, Navitip, jiffy tube, and local anesthetic syringe (Table 3).

**Table 3:** Full description and characteristics of the included in-vitro studies

**Study**	**Country**	**Material**	**Technique**	**Operator**	**Last file size**	**File type**	**N**	**Overfill** **(%)**	**P value**	**Unit of outcome**	**Tooth type**	**Filing distance from apex (mm)**	**Radiographic assessor**	**Inter-examiner reliability**
Guelman, 2004^2^	USA	ZOE	Handpiece lentulo	NI	40	NI	15	0 (0)	0.22	Canal	Incisor	1-2	Double-blinded and calibrated	No
			Navitip				15	3 (20)						
		Vitapex	Handpiece lentulo				15	3 (20)	0.5					
			Provided syringe				15	1 (7)						
Hiremath, 2016^30^	India	ZOE	Pressure syringe,	NI	35	K	24	0 (0)	0.05	Canal	Molar	1	Double-blinded and calibrated	No
			Insulin syringe				24	4 (17)						
			Jiffy tube				24	1 (4)						
			Anesthetic syringe				24	5 (21)						
Memarpour, 2013^3^	Iran	ZOE	Handpiece lentulo	NI	35	K	43	10 (23)	.43	Canal	Molar	1	Double-blinded and calibrated		No
			Plugger with cotton pellet				39	7 (18)							
			Anesthetic syringe				37	4 (11)							
			NaviTip syringe				38	3 (8)							
			Pressure syringe				39	6 (15)							
			Tuberculin syringe				43	5 (12)							
Walia, 2017^31^	UAE	ZOE	Handpiece lentulo	NI	35-40	H	15	2 (13)	0.48	Tooth	Molar	1	Double-blinded and calibrated	Yes
			Lentulo				15	0 (0)						
			Vitapex				Provided syringe	15							8 (53)	

ZOE: Zinc-Oxide Eugenol; NI: not indicated; RCT: randomized clinical trial

### Quality assessment

Only two studies^19,32^ presented sample size calculation, one of which had explicitly compared different techniques.^19^

In the clinical studies, root canal treatments were carried out by a pediatric dentist in four studies and by postgraduate students in two studies. The remaining clinical studies and all of the in vitro studies did not mention the operator of root canal filling procedures (Table 1).

For the radiographic assessment of treatment outcome, 15 studies employed at least two observers to carry out the assessment. The observers were calibrated before evaluating radiographs in nine studies, and inter-observer reliability tests were carried out in seven studies (Table 1).

None of the included studies were categorized as having a low risk of bias. Eleven studies had a high risk of bias. In 13 studies, the risk of bias was unclear. The details are presented in Table 4.

**Table 4:** “Risk of bias” summary table for included studies

	**Random sequence generation** **(selection bias)**	**Allocation concealment** **(selection bias)**	**Blinding of outcome assessment (detection bias)**	**Incomplete outcome data** **(attrition bias)**	**Overall risk of bias**
**Clinical studies**					
Ann Mani, 2000 ^46^	-	-	?	+	High
Barcelos, 2011 ^32^	+	?	+	+	Unclear
Bawazir, 2005 ^20^	+	?	+	+	Unclear
Chawala, 2008 ^10^	NA	NA	-	+	High
Damle, 2005 ^47^	?	?	?	+	Unclear
Gandhi, 2017 ^28^	?	?	+	+	Unclear
Grover, 2013 ^18^	?	?	+	+	Unclear
Gupta, 2011 ^48^	-	-	-	+	High
Khubchandani,2017 ^29^	?	?	+	+	Unclear
Louwakul, 2012 ^52^	+	?	?	+	Unclear
Mortazavi, 2004 ^11^	+	?	-	+	High
Nakornchai, 2010 ^53^	+	?	-	+	High
Ozalp, 2005 ^17^	?	?	-	+	High
Pandranki 2017 ^27^	?	?	+	+	Unclear
Ramar, 2010 ^49^	?	?	-	+	High
Rewal, 2014 ^50^	?	?	?	+	Unclear
Sarý, 2008 ^54^	NA	NA	-	+	High
Subramaniam, 2011 ^40^	?	?	-	+	High
Tannure, 2010 ^51^	+	?	-	+	High
Vashista, 2015 ^19^	-	-	+	+	High
**In-vitro studies**	
Guelman, 2004 ^2^	?	?	+	+	Unclear
Hiremath, 2016 ^30^	?	?	+	+	Unclear
Memarpour, 2013 ^3^	?	?	+	+	Unclear
Walia, 2017 ^31^	?	?	+	+	Unclear

NA: not applicable

### Overfilling of ZOE with different techniques in clinical studies

Overfilling of ZOE was reported in 13 studies in which a hand-held lentulo spiral (four studies), plugger (three studies), pressure syringe (two studies), lentulo spiral mounted on a handpiece (six studies), and Navitip (one study) were used for root canal obturation. Three studies explicitly compared different techniques. Vashista et al^19^ compared a hand-held lentulo spiral with a pressure syringe, Bawazir and Salama^20^ compared a lentulo spiral mounted on a handpiece with a hand-held lentulo spiral, and Khubchandani^29^ compared a lentulo spiral mounted on a handpiece with Navitip (Table 1). No significant differences were reported between the techniques in the rate of overfilling in the studies above. The meta-analysis of two-by-two comparisons was not applicable because of the inadequate number of studies.

A meta-analysis was performed to estimate each technique’s overfilling rate in these 13 studies, and no critical heterogeneity was detected.

**Figure 2:**
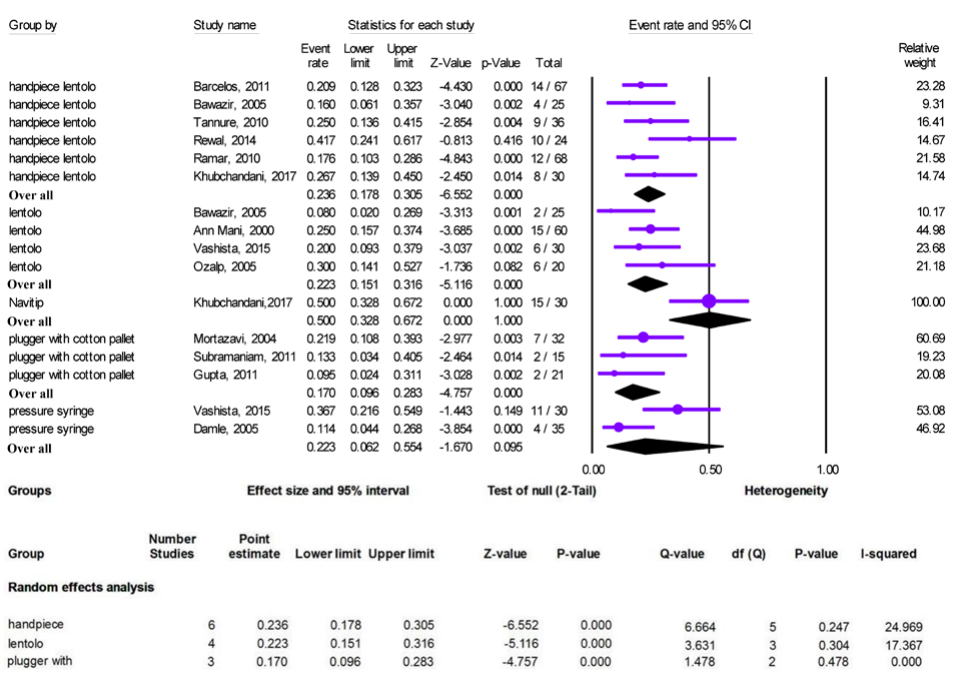
Forest plot and meta-analysis of pulpectomy overfill rate from clinical studies that used different techniques with zinc oxide-eugenol.

The random-effects meta-analysis revealed that the pooled overfilling rate of ZOE was 23.6% (95% CI: 17.8‒30.5) with a lentulo spiral mounted on a handpiece, 22.3% (95% CI: 15.1‒31.6) with a hand-held lentulo spiral, and 17% (95% CI: 9.6‒28.3) with plugger and cotton pellet techniques. The Navitip and pressure syringe techniques were not included in the meta-analysis because of the inadequate number of studies (Figure 2).

### Overfilling of calcium hydroxide-based materials with different techniques in clinical studies

Overfilling of calcium hydroxide-based materials was reported in 15 studies in which a hand-held lentulo spiral (four studies), plugger with cotton pellet (two studies), pressure syringe (two studies), provided syringe (six studies), a lentulo spiral mounted on a handpiece (four studies), Navitip (one study), a disposable syringe (one study), and Pastinject (two studies) were used for root canal obturation. Three studies compared the different techniques. Grover et al^18^ compared a lentulo spiral mounted on a handpiece, pressure syringe, bi-directional spiral, and Pastinject for Endoflas and reported a significantly higher number of overfilled canals with the pressure syringe. Pandranki et al^27^ compared a lentulo spiral mounted on a handpiece, Navitip, and plugger with cotton pellet for Endoflas and reported no significant differences between the techniques. Gandhi et al^28^ compared disposable syringe, a hand-held lentulo spiral, and Pastinject for Endoflas and reported no significant differences in the overfilling rate between the techniques (Table 2). The meta-analysis of two-by-two comparisons was not applicable because of the inadequate number of studies.

**Figure 3:**
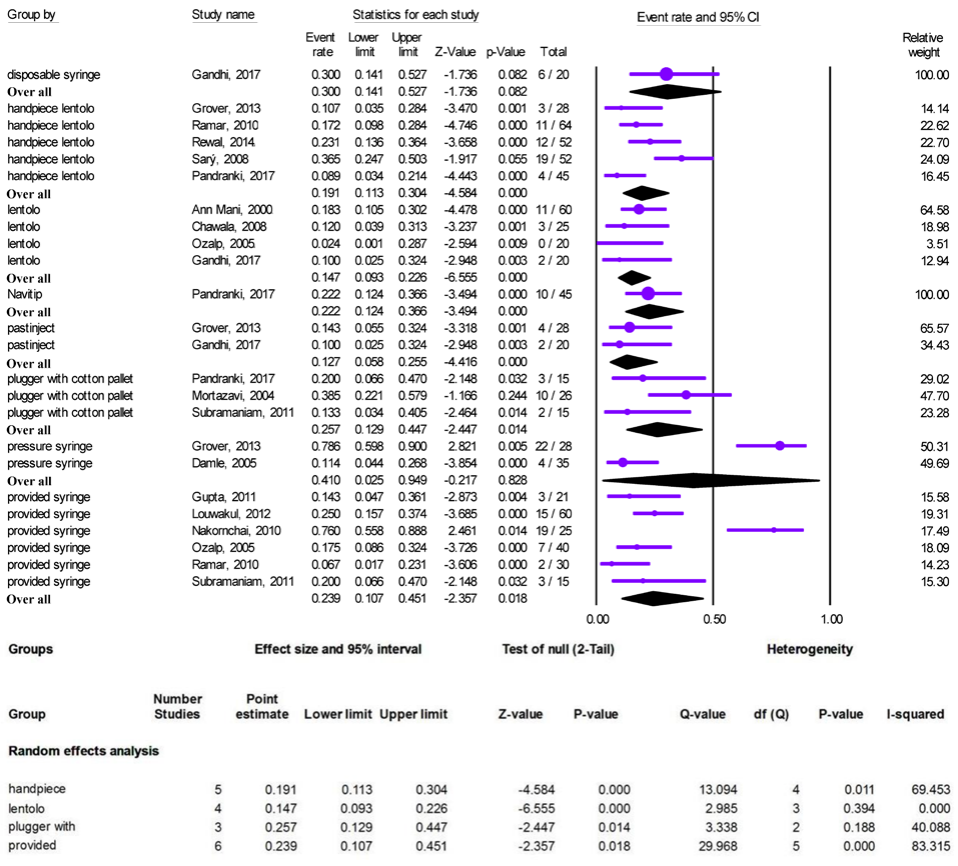
Forest plot and meta-analysis of pulpectomy overfill rate from clinical studies that used different techniques with calcium hydroxide-based materials.

A meta-analysis was performed to estimate the overfilling rate with each technique used in these 15 studies. There was substantial heterogeneity in the provided syringe (Q-value=29.968, I^2^=83.315) and a lentulo spiral mounted on a handpiece (Q-value=13.094, I^2^=69.453), and no critical heterogeneity in plugger and cotton pellet technique (Q-value=3.338, I^2^=40.088). No heterogeneity was detected in the lentulo spiral technique (Figure 3).

The pooled overfilling rate of calcium hydroxide-based materials derived from the random-effects analysis was 19.1% (95% CI: 11.3‒30.4) with a lentulo spiral mounted on a handpiece, 14.7% (95% CI: 9.3‒22.6) with a hand-held lentulo spiral, 25.7% (95% CI: 12.9‒44.7) with plugger with a cotton pellet, and 23.9% (95% CI: 10.7‒45.1) with the provided syringe techniques. Pressure syringe, Navitip, Pastinject, and disposable syringe techniques were not included in the meta-analysis because they comprised less than three studies (Figure 3).

### Overfilling with different techniques in vitro

Diverse and not overlapping techniques were investigated in the included in vitro studies. Therefore, neither individual overfilling rates nor two-by-two comparisons were subjected to meta-analysis.

Memarpour et al^3^ compared a lentulo spiral mounted on a handpiece, plugger with a cotton pellet, and four injection techniques for ZOE and reported no significant differences in the overfilling rate. Guelmann et al^2^ reported no significant difference between Navitip and a lentulo spiral mounted on a handpiece for ZOE. Hiremath and Srivastava^30^ observed no significant differences between the four injection techniques for ZOE. Walia et al^31^ reported no significant difference between a hand-held lentulo spiral and a lentulo spiral mounted on a handpiece for ZOE. In addition, Guelmann et al^2^ reported no significant difference between lentulo mounted on a handpiece and the provided syringe for Vitapex. Detailed descriptions of the included in vitro studies are presented in Table 3.

## Discussion

Various root canal obturation techniques and materials are used to adequately adapt the paste to root canal walls, completely fill the root canal, and acquire an optimum apical seal without overfilling, which are major predicting factors for preventing recurrence of bacterial infection and successful root canal treatment of primary teeth.^1,22-25,29,33-36^ This review was undertaken for the first time to evaluate the overfilling rate of primary tooth pulpectomy by different placement techniques. Observational and cross-sectional studies, clinical trials, and in vitro studies that presented useful data were included, although they had no randomization or control groups.

Overall, the quality of evidence and the methods used to record and report the outcomes in the existing studies are not optimal. There are significant variations in study protocols and treatment procedures, and even in the current treatment guidelines, which significantly impact the outcomes of root canal treatment and make it unfeasible to investigate the effect of individual clinical factors on the quality of root canal treatment in primary teeth. There were also differences between the studies in the radiographic criteria for the extent of root canal obturation, the unit of outcome measure (canal and tooth), type of treated tooth, and filing distance from the apex (working length). There is a need for consistency in design, data collection, reporting and evaluating treatment results, and establishing gold standard treatment guidelines to control the dominant factors influencing treatment outcomes.

The definition of optimal obturation is not strict in the current literature and has been considered to be up to 2 mm short of the apex,^20,31^ within 0‒1.5 mm of the apex,^3^ less than 1 mm short of the apex^32^ or flush-filled.^37^ This inconsistency does not allow the accurate extraction of the rate of optimal or underfillings with different obturation techniques. In addition, all the teeth with “any” canal showing the extrusion of filling material have been considered to be overfilled in the included studies even if the other canals had optimum obturation or underfillings.^19,20,31,32^ Therefore, the use of tooth as the unit of outcome measure is not recommended since it undermines the accuracy of assessments and leads to the overestimation of overfilling or underestimation of optimal and underfilling rates.

The complex anatomy of the primary molar root canals is well established. Narrow and ribbon-shaped canals, lateral branching or fusion of canals, and apical resorption make adequate root canal cleaning and shaping difficult.^38,39^ The majority of included studies were conducted on posterior teeth. However, five studies had a mixed sample comprising both anterior and posterior teeth, and two studies included only anterior teeth. This factor might influence the pooled overfilling rates because anterior teeth have straight canals and less complexity.

Other factors than the root canal filling technique might also increase the chance of overfilling. The existence of radicular pathological lesion, thin dentinal walls in the inter-radicular areas, physiological or pathological resorption of the bone and root apex, wide and straight canals, extensive preparation of canals, and thin consistency of the filling material can facilitate the extrusion of the filling material.^20,28,29,31,40,41^

The educational status and experience of the operators also impact their performance and the quality of treatments. A prior meta-analysis revealed that the success of root canal treatment by endodontists or postgraduate students was higher than in other dentist groups.^42^ Therefore, the successful outcome of studies in which a single operator carries out all the treatments might also be related to superior operator skills rather than a superior technique because the techniques are operator-sensitive.^28^ In the present review, most of the included studies did not mention the operator of root canal filling procedures. Although it is not possible to objectively quantify operator skills, future studies should consider operator skills and also report the qualifications of the operators who performed the treatments.

The main and the most critical shortcoming of the included studies, which significantly compromises the validity of the outcomes, was that all the studies had a small sample size. Surprisingly, only one of the included studies^19^ that aimed to compare different techniques had sample size calculation. Unfortunately, this factor was missing even in the most recent studies, which is below the current standards and guidelines. Future research in this field should consider representative sampling, recruitment standardization, and justification of sample size to ensure the study’s sufficient power to detect differences.

In addition, the included studies had an unclear or high risk of bias as they failed to record some information considered essential for bias-free reports. In some cases, this was due to incomplete reporting of study procedures rather than the actual design and implementation of the study. A significant source of the lack of clarity was the allocation of study participants or samples and allocation concealment. Although most studies mentioned that the allocations were random, it was not clear whether it was implemented appropriately. Therefore, designing and reporting of studies in this field need to be improved to secure obtaining scientific evidence and the reliability or relevance of the findings. It has been shown that studies, in which randomization and allocation concealment procedures were inadequate, tended to overestimate treatment effects. In addition, calibrated and ideally blinded examiners not involved in the treatment procedures should carry out the outcome assessments. Blinded evaluation is necessary to prevent overestimation of treatment effects.^43,44^

The meta-analysis results revealed that the lowest rate of overfilling of ZOE in the clinical studies was with plugger and cotton pellet (17%). The hand-held lentulo spiral technique had the lowest overfilling rate (14.7%) when used for calcium hydroxide-based materials in clinical studies. The discrepancy between the results is probably due to the differences in the consistency of filling materials, type of teeth, sample size, tip thickness of filling instruments, operator experience, and mainly the fact that the unit of outcome measure was different between the calcium hydroxide-based materials and ZOE groups. In addition, because of the limited number of in vitro studies, conclusive interpretation and meta-analysis of in vitro results were not possible.

Lentulo spiral was the most used instrument for root canal obturation in primary teeth. Its design and flexibility allow easy filling of both straight or narrow and curved root canals in primary teeth. However, it does not produce a densely compacted root canal filling, and much reliance is placed on the adherence of the paste to the root canal walls.^28^ Difficulties with fixing the rubber stop, instrument fracture, and the need for repeated removal and reinsertion of the instrument and consequently formation of voids are significant disadvantages of the lentulo spiral. The reason for overfilling with the lentulo spiral might be related to the loss of operator feel and displacement of the rubber stop during the filling procedure.^3^ However, the operator might have more tactile sensation with a hand-held lentulo spiral than a lentulo spiral mounted on a handpiece.^31^ Pastinject is an instrument similar to a lentulo spiral, which was used in two studies. It has flattened blades reported to improve material placement into the root canal and obturation quality.^28^

On the other hand, the thicker tip of the plugger and its limited flexibility make it difficult to reach the apex, especially in curved or narrow canals. However, this method has high efficacy in long, straight canals, such as those of primary anterior teeth. The movements of the plugger during paste application might increase the formation of large voids and the rate of overfilling, especially when the material has thick consistency or the instrument has thin tip.^3^

Of the available injection techniques, ZOE was used with a pressure syringe, and calcium hydroxide-based pastes were used with their provided syringe, NaviTip or disposable syringe, and pressure syringe in clinical studies. Anesthetic syringe, tuberculin syringe, insulin syringe, and Jiffy tube were only used in the in vitro studies. The NaviTip is a highly flexible thin metal tip with different sizes designed to deliver the paste and sealer into the root canal.^45^ The tip increases the operator feel during injection and can penetrate the curved, narrow root canals close to the apex and inject paste rapidly and uniformly.^3,29^ However, the needle used in the remaining injection techniques is not as flexible as the NaviTip and does not reach the apex in curved canals.^3^

Injection techniques have a general defect. The amount of appropriate pressure for adequate filling of the canals cannot be estimated by the operator. Therefore, the risk of overfilling increases with these techniques, particularly when the operator is inexperienced and applies excessive injection pressure, the material has a loose consistency, the tooth has wider apical foramina or extensive canal preparation, or the needle reaches the root apex.^18,28,31^ The displacement of the rubber stop, the need for repeated removal of the needle to refill the syringe during the procedure, and difficulty separating the needle might create voids, over-push the paste, and decrease the obturation quality. In addition, it is needed to immediately clean the syringe after use with some of these techniques.^3,29^

Another factor that determines the quality of root canal filling and its success is the presence of voids. Voids might lead to leakage in the paste, facilitating micro-organism regrowth, reinfection, and an increased risk of post-treatment disease, especially if there are several large voids. Factors that influence the location and size of the voids include the type, viscosity, and consistency of the paste, the method used to apply the paste, and operator skill and experience.^3^ Air bubbles might be entrapped in the paste during mixing of the powder with the liquid and during repeated removal and reinsertion of the instrument in the filling procedure.^3^ In addition, void formation might increase with pressure syringe and insulin or tuberculin syringe if air enters the cartridge when it is filled.^3,38^ The efficacy of different techniques to achieve void-free obturation needs to be investigated in future studies.

## Conclusion

Based on the findings of this study and within the limitations of available data, the following conclusions can be drawn:

1. The lowest overfilling rate for ZOE in clinical studies was related to using a plugger with a cotton pellet.
2. The lowest overfilling rate for calcium hydroxide-based materials in the clinical studies was related to a hand-held lentulo spiral.

## Authors’ Contributions

NAA contributed to the concept, data acquisition and interpretation, and critically revised the manuscript. ZJ contributed to data acquisition and drafted the manuscript. SS contributed to the concept and design, data acquisition, analysis, and interpretation, and drafted and critically revised the manuscript. All the authors approved and agreed to be accountable for all the aspects of the work, ensuring integrity and accuracy.

## Funding

This study was supported and funded by the Dental and Periodontal Research Center.

## Competing Interests

The authors declare that they have no competing interests and no conflict of interests.

## Ethical Approval

This article does not contain any studies with human participants or animals performed by any of the authors.
